# Design and Implementation of ESP32-Based IoT Devices

**DOI:** 10.3390/s23156739

**Published:** 2023-07-27

**Authors:** Darko Hercog, Tone Lerher, Mitja Truntič, Oto Težak

**Affiliations:** 1 Faculty of Electrical Engineering and Computer Science, University of Maribor, Koroška Cesta 46, 2000 Maribor, Slovenia; mitja.truntic@um.si (M.T.); oto.tezak@um.si (O.T.); 2 Faculty of Logistics, University of Maribor, Mariborska Cesta 7, 3000 Celje, Slovenia; 3 Faculty of Mechanical Engineering, University of Maribor, Smetanova Ulica 17, 2000 Maribor, Slovenia; 4 Technical School Center Maribor, Zolajeva Ulica 12, 2000 Maribor, Slovenia

**Keywords:** ESP32, Arduino, IoT, education, analog discovery

## Abstract

The Internet of Things (IoT) has become a transformative technology with great potential in various sectors, including home automation, industrial control, environmental monitoring, agriculture, wearables, health monitoring, and others. The growing presence of IoT devices stimulates schools and academic institutions to integrate IoT into the educational process, since IoT skills are in demand in the labor market. This paper presents educational IoT tools and technologies that simplify the design, implementation, and testing of IoT applications. The article presents the introductory IoT course that students perform initially and then presents some of the projects that they develop and implement on their own later in the project.

## 1. Introduction

The Internet of Things (IoT) is a network of devices, users, and services; their connectivity enables sensing, decision-making, and reactions [1]. In 2022, there were 14.3 billion active IoT devices connected to the network, a number estimated to grow 16% this year to 16.7 billion active points. The number of connected devices will increase even further in the coming years. The forecast for 2027 is 29.7 billion active devices [2]. IoT technology solutions enable remote monitoring and operation of industrial environments [3], and also facilitate the monitoring of unconnected remote areas [4]. According to [5,6], there are four essential characteristics of a device to call it a thing in IoT terms:

- The device must be able to acquire data from its surroundings and transmit it to other devices or directly to the Internet.
- The device must be able to react according to the current circumstances.
- The device must be able to receive information from the network.
- The device must support communication; IoT devices inherently belong to a network of devices that communicate with each other through nodes on the same network.

IoT devices support three layers:

- The physical layer, which comprises sensors and actuators;
- The network layer, which interconnects the devices via networks all the way to the users;
- The application layer, which connects physical and network layers digitally and facilitates interactions between the user and other machines, thus allowing people to make decisions at a higher level of control or to automate selected tasks.

IoT devices are used in a wide range of applications, including (1) home automation, (2) industrial monitoring and control, (3) environmental monitoring, (4) agriculture and farming, (5) wearables and health monitoring, (6) smart energy management, and (7) data logging and analytics. In the home automation field, IoT devices control and automate various household devices, including lights, thermostats, door locks, security cameras, and home appliances [7]. In home automation applications, IoT devices are typically connected to Wi-Fi networks and communicate with cloud platforms, thus allowing users to monitor and control household appliances remotely [8,9]. Robotics, automation, and industrial monitoring are other fields where IoT devices are employed usefully for real-time control and monitoring of equipment, machinery, and processes [10]. IoT devices collect sensor data, exchange data with other devices using industrial protocols, and interact with control systems to optimize operations, enhance machinery efficiency, and enable predictive maintenance to be carried out. In robotics, IoT devices acquire data and control robot motors and other actuators [11]. Environmental monitoring is an area where IoT devices are widespread. IoT devices are used for environmental monitoring, such as weather stations [12], monitoring air quality [13,14], temperature [15], humidity, and pollution levels [16]. In most applications, IoT devices collect data, analyze them, and send them to cloud platforms for further analysis and visualization. IoT devices can also be found in agriculture and farming systems, where they increase farm productivity. IoT devices monitor and automate agricultural processes, collect data from soil moisture sensors, weather stations, and other sensors in order to optimize automated irrigation systems [17,18], control water levels in water tanks [19], facilitate the control of greenhouse environments, facilitate crop management, and enable the tracking of livestock. Compact size and low-energy consumption IoT devices are used in wearables and health monitoring applications. They are used in fitness trackers, smartwatches, and medical devices to collect data such as heart rate, body temperature [20,21], and physical activity. The collected data can be analyzed locally or transmitted to cloud platforms for further processing. IoT devices could also be used beneficially in rehabilitation processes [22] and safety monitoring applications [23]. IoT asset tracking devices are used mainly in logistics and inventory management to track the location and motion of objects or vehicles. Through the integration of IoT devices with GPS modules and sensors, assets can be tracked in real time and a variety of parameters, such as monitoring acceleration, speed, temperature, and humidity, can be monitored. IoT asset tracking devices enable the real-time tracking, monitoring, and optimization of goods, vehicles, or equipment within warehouses or supply chains. Smart energy management IoT systems are used to monitor and control energy usage. They collect data from smart meters, monitor energy consumption patterns, and enable the remote control of devices to optimize energy efficiency and reduce costs [24,25]. Given the above, the IoT has become a technology with great potential in various sectors, including education. The IoT is being used increasingly in education to enhance student engagement, student satisfaction, and the quality of learning [26]. IoT applications in academia provide new opportunities for innovation, and improvements in the learning process and educational institutions’ infrastructure [1]. The impact of the IoT on education can be observed in terms of sensors and data availability, as well as smart living, working, and classroom environments. The increasing omnipresence of IoT devices encourages schools and academic institutions to integrate IoT into educational activities [27], thus aiming to support the pedagogic process for all three parties: (1) institutions, (2) students, and (3) teaching staff.

This article examines educational activities relating to the IoT, focusing on using sensors and technologies in educational settings and developing IoT-related educational projects. The article presents the IoT tools and technologies we use to teach mechatronics students. This paper presents self-developed hardware used in the initial phase of teaching IoT concepts. The article also presents the content of the introductory IoT course that students perform initially and then presents some IoT prototypes that they develop and implement later in the project.

The article is structured as follows. Related works are presented in Section 2. Section 3 presents the used software and hardware. This chapter also describes the IoT training and testing process briefly using the presented hardware and software. Section 4 presents some of the IoT prototype devices designed and developed by students. Summaries and some pointers to future work are provided in the conclusion.

## 2. IoT in Education

The use of advanced IoT technologies is essential for application-oriented universities (Xie, 2021, [27]; Mircea, 2021, [28]; Yawson, 2018, [29]; Gul, 2017, [1]; Zhou, 2020, [30]; Pervez, 2018, [31]); it not only directly affects students but also, and above all, educational providers. According to research [32], the most common ways of integrating IoT devices into the educational process are as follows:

- A smart environment to support learning;
- E-learning, teaching, and distance education management;
- Remote labs;
- Augmented reality learning and distance learning;
- Wireless robotic learning platforms;
- Distance attendance management systems;
- An IoT-enabled education service.

IoT technology can improve the energy efficiency of a school building [33], enhance the learning process, or make the lecturer’s job easier. The idea of a smart lecture room realized with the IoT is presented in [34]. Tan [35] proposed to improve the learning process through RFID technologies that facilitate the continuous monitoring of students’ attendance and work. In [36], a laboratory is presented where IoT technology is used to teach microprocessor technology by monitoring the microcontroller’s pins with sensors and, through a monitoring system, communicating to the professor the student’s progress during learning. In this way, learning outcomes are improved. The IoT enables the connection between information and communication technologies. Paper [37] discusses the use of IoT devices for monitoring and evaluating the quality of the teaching process from the perspective of managing study processes and institutions. Data on student and lecturer behavior collected by IoT devices using sensors are collected through the communication layer, and they are sorted and analyzed using data mining. The described procedures aim to achieve a harmonious relationship between students and lecturers to maximize learning outcomes.

Introducing the IoT in education brings previously unfeasible approaches and solutions through gamification [38]. The authors [38] identified opportunities to develop innovative virtual labs and interactive student experiences. The IoT offers a near real-time visualization of the real world. The data from the sensors are transmitted over a network to computer systems that filter and analyze the data. The result is a realistic and safe simulation environment that offers students a new learning experience. Gamification can be used in classrooms or for distance learning. It facilitates the delivery of study content and increases students’ motivation to learn. In addition, gamification and virtual labs also mean a reduction in equipment costs. High-quality simulators can train people, machines, and artificial intelligence models.

A report by Curtin University [39] identifies the relationship between technology and tertiary education for students with disabilities. They are focused on the pedagogical implications of using IoT by students with disabilities who use mobile devices as critical tools in their studies. They conclude that IoT devices offer more significant potential for including students with disabilities in tertiary education, and a better learning experience for all students, due to the possibility of customization for the individual student. The possibility of making smaller devices that do not require much power and that can often be controlled by smartphones offers new opportunities for student participation in the automation and remote control of devices. The recommendations can be summarized in the following conclusions: (1) the integration of IoT devices into the learning process should be planned carefully; (2) IoT devices should be integrated into specific pedagogical content; (3) students should also be able to access IoT devices through their phones; (4) the implementation of IoT devices should also take into account security and privacy aspects; (5) any integration of IoT devices should be supported by appropriate training, so that staff and students can make effective use of the technology’s potential; (6) IoT devices can also be used as effective digital assistants for students with disabilities.

IoT technologies are being deployed as part of the pedagogical process in the following ways:

- For delivering teaching, to support students, lecturers, and management staff;
- For establish the content (subject) of the study, which allows students to play an active role. This includes the presented realization, which is described below.

The authors Song and Meng [40] and Nikolayev [41] noted that a project-based teaching method, where lecturers are only in the role of guiding and assessing student work, improves the quality of teaching. Technologies like IoT are bound to impact the education system and offer opportunities to implement grassroots transformation [41], including transport systems, robotics [42], control [43], and teaching [35,44,45,46,47]. Luis Bustamante et al. [48] proposed an open-source platform for collecting, managing, and analyzing large amounts of sensor data. In [46], the authors presented an IoT module with artificial intelligence features. A module with an STM32 chip handles multiple sensors, implements a fuzzy algorithm, performs machine learning and weight adjustment, and controls I/O communication. Carlos-Mancilla et al. [49] described the design and development of an IoT weather station. It measures eight weather quantities and uploads the data to the cloud. In [42], the authors presented the design and development of an affordable open-source robotic arm for online robotics teaching during a pandemic. The robotic arm is based on an ESP32 microcontroller, and it can be controlled remotely using a smartphone app developed with the Blynk application. The presented robotic arm is an inexpensive and reproducible project that helps students learn robotics’ design through hands-on experience. The paper also provides details of the robotic arm’s components, direct and inverse kinematics, associated software, simulation, and experimental results. A low-cost multi-agent system experimental platform for teaching and research of decentralized cooperative control is presented in [43]. The platform consists of autonomous agents (small cars) equipped with an ESP32 module, a time-of-flight (ToF) ranging sensor (VL53L0X), an optical flow sensor (ADNS3080), LEGO motors, an H-bridge (TB6612FNG), and a 3D-printed chassis. The platform can be used to present essential topics in control engineering education. Individual agents can be used for simple PID experiments in a classroom, while a collection of agents can perform decentralized platooning with cooperative adaptive cruise control. The platform is constructed from low-cost components and programmed with open-source software, making it affordable and valuable for students and researchers. Setiawan et al. [50] proposed a Kobela teaching aid for teaching multiplication and division in mathematics to second-grade primary school students. The Kobela can improve students’ concentration, creativity, and learning outcomes. Kobela includes an Arduino Uno microcontroller, a Wi-Fi module (ESP8266-01), a keypad, a color sensor, and a buzzer. Kobela uses IoT technology to read, assess, and record learning activities. An open-source hardware platform that implements a Wi-Fi-supported RFID reader is presented in [35]. The proposed system assists teachers in performing the automatic collection of attendance records and student behavior records.

The presented IoT system improves student attendance and impacts their learning process positively. In [44], a university IOT course is presented with an example of two IoT projects. The first project presents a weather station based on the Arduino Uno microcontroller and the DHT22 sensor. The second project is based on the Arduino Esplora board, and it allows remote users to observe/control Esplora sensors/actuators using a developed web page. An IoT-based method for teaching sensor technologies in Greece’s VET (Vocational Education and Training) program is presented in [47]. The proposed solution comprises a theoretical approach and a laboratory practice, utilizing existing laboratory equipment and the Arduino platform. IoT technologies are considered essential for learning in the field of sensors. Kleinschmidt [51] presented an undergraduate course teaching IoT concepts for engineering students using collaborative problem-based learning. The students develop a project using IoT technologies: sensors and actuators, embedded computing, communication and networking, cloud computing, and data analysis. Several projects are presented in the article (Smart Dispenser Automatic Frequency and Localization Control, Smart Traffic Light, Comfort Room, iQuarium) that, in most cases, are based on NodeMCU (ESP8266 chip) and Raspberry Pi microcontrollers and the ThingSpeak and Konker web platforms. The development and architecture of the remote IoT lab are described in [52]. The distributed computing environment facilitates integration between students’ smartphones and IoT devices in the campus labs. The remote lab was developed for the digital electronics lab to enable students to run experiments during the COVID-19 pandemic. In [45], the authors presented effective teaching using a real-time water quality monitoring prototype based on a Texas Instruments 32-bit ARM Cortex-M4 microcontroller, ESP8266 Wi-Fi module, temperature, and a pH sensor. A prototype analyzes and transmits real-time water quality parameters to a server.

## 3. Materials and Methods

This section introduces the software and hardware used in the introductory IoT course.

### 3.1. Hardware

#### 3.1.1. ESP32

ESP32 [53] is a powerful and cost-effective platform for developing IoT applications. The ESP32, developed by the Espressif Systems Company (Shanghai, China), offers a powerful combination of features and capabilities for IoT applications. ESP32 has the following features: (1) a dual-core processor, (2) integrated Wi-Fi and Bluetooth connectivity, (3) a large number of general purpose input/output (GPIO) pins, and (4) low power consumption. ESP32 is equipped with a dual-core Tensilica (Santa Clara, CA, USA) Xtensa LX6 microprocessor, which provides higher processing power and facilitates multitasking and efficient execution of complex tasks. ESP32 has built-in Wi-Fi and Bluetooth interfaces that simplify connection and communication with other devices or networks. It supports various Wi-Fi protocols, such as 802.11 b/g/n, and it provides Bluetooth Classic and Bluetooth Low Energy (BLE) connectivity options. The ESP32 provides many GPIO pins that facilitate connection with and control of external devices and sensors. These pins support a variety of interfaces, including SPI, I2C, UART, and PWM. The ESP32 is designed to be power efficient, thus enabling the development of energy-efficient IoT applications. It offers sleep modes and power management features that help reduce power consumption, making it suitable for battery-powered or energy-constrained projects. ESP32 can be connected to displays, touchscreens, or LED indicators to provide operators or staff with a user-friendly interface.

The ESP32 can be programmed using various development frameworks and languages. The most commonly used programming language is C++, and it can be programmed using the Arduino IDE or PlatformIO. In addition, the ESP-IDF (Espressif IoT Development Framework) provides a comprehensive set of libraries and tools specifically for ESP32 development.

**Figure 1:**
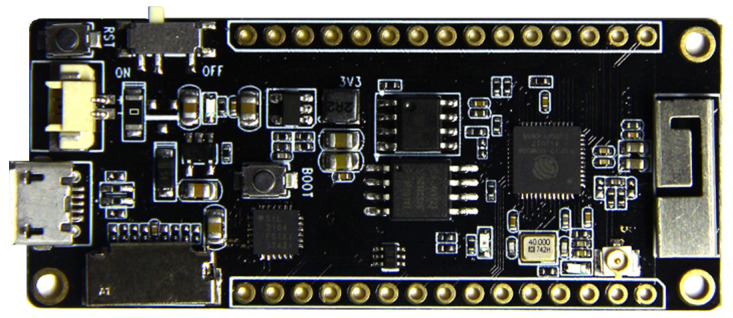
LilyGO TTGO T8 module.

Several modules on the market are based on the ESP32 chip. Some modules come with built-in sensors, simplifying the integration of these sensing capabilities into IoT projects. We decided to use the TTGO T8 from the LilyGO module (Figure 1), as it is one of the rare models on the market that allows the user to connect an SD card. As a result, the module is suitable for applications involving data logging. The module includes a micro-USB connection that can be used for programming or power. In addition, the module includes the following features:

- Processor: ESP32 (24 MHz dual core);
- Flash memory: 4 MB;
- Built-in microSD card connector;
- PSRAM (pseudo-static random access memory): 8 MB;
- Built-in Wi-Fi, Bluetooth, USB to serial converter (CP2104 or CH9102F);
- Built-in Li-ion/Li-Po battery charging circuit: TP4054 chip.

#### 3.1.2. Analog Discovery 2

**Figure 2:**
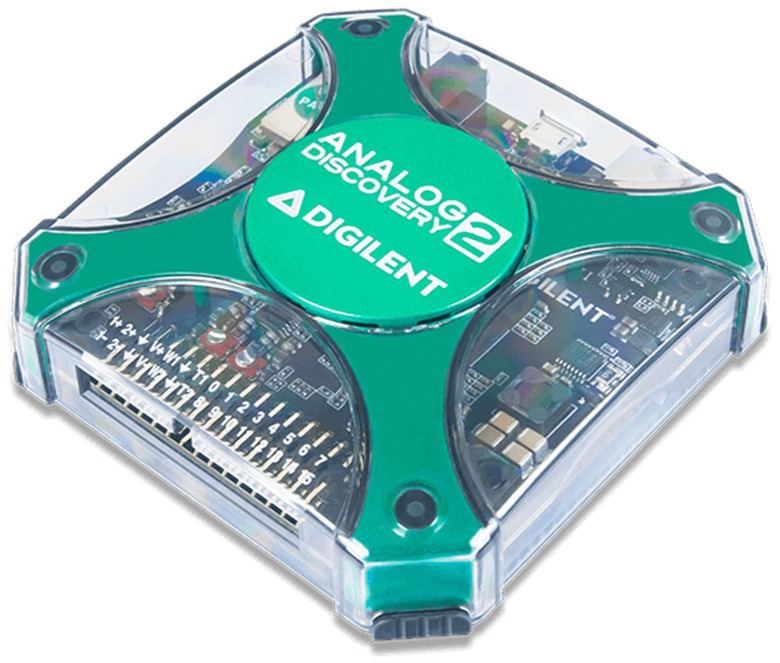
Digilent Analog Discovery 2.

Analog Discovery 2 [54] (Figure 2) is a USB-powered test and measurement device developed by the Digilent company (Pullman, WA, USA). Analog Discovery 2 represents a versatile tool for electronics hobbyists and professionals involved in circuit design, prototyping, and testing. This device combines multiple functionalities into a single compact device, providing a cost-effective and portable solution for various measurement and analysis tasks. The Analog Discovery device is accompanied by Digilent WaveForms software, which provides an intuitive user interface for controlling and visualizing the measurements and signals. Analog Discovery 2 includes an (1) oscilloscope, (2) waveform generator, (3) power supply, (4) logic analyzer, (5) pattern generator, (6) spectrum analyzer, and (7) data logger. The 2-channel oscilloscope enables a maximum sampling rate of 100 MS/s. The waveform generator is capable of producing various standard and custom waveforms. It gives the user precise control over frequency, amplitude, and waveform shape, and it is usually used for testing and stimulus purposes. In addition, Analog Discovery offers two programmable power supplies, with adjustable voltage outputs for powering external circuits or components. The device also has a built-in logic analyzer that captures and analyzes digital signals with up to 32 channels. Analog Discovery also includes a pattern generator for generating digital patterns and simulating digital circuits. The device also provides a spectrum analyzer that enables basic spectrum measurements of input signals. Finally, Analog Discovery can also be used as a data logger since it enables capturing and recording data from various sources over time.

#### 3.1.3. Custom Interface Boards

IoT applications use various chips that measure physical quantities such as temperature, humidity, and pressure. These chips convert the measured quantities into digital form and then transmit the information to the processing unit using analog or digital signals. More advanced chips typically use communication buses to exchange the data, like 1-Wire, UART, I2C, and SPI. In order to establish a connection between the sensor chip and the processing unit, a particular printed circuit board needs to be developed; however, this process is time consuming. In most cases, using a commercially available module with the appropriate sensor chip is a better and more rapid way to test it, since custom printed circuit boards (PCB) design, soldering, or complicated wiring is unnecessary. Several market vendors, like Adafruit (New York, NY, USA), Seeed Studio (Shenzhen, China), and SparkFun (Boulder, CO, USA), produce modules with the most commonly used chips. However, these manufacturers use different connectors, so an appropriate expansion board is necessary to connect the sensor modules to the processing unit.

Custom interface PCBs have been designed and developed in order to connect the LilyGO TTGO T8 module to commercially available sensor/actuator modules and an Analog Discover instrument. The developed ESP32 sensor extension board enables connection of Grove (Seeed Studio)-, STEMMA QT (Adafruit)-, and Qwiic (SparkFun)-based sensor/actuator modules.

**Figure 3:**
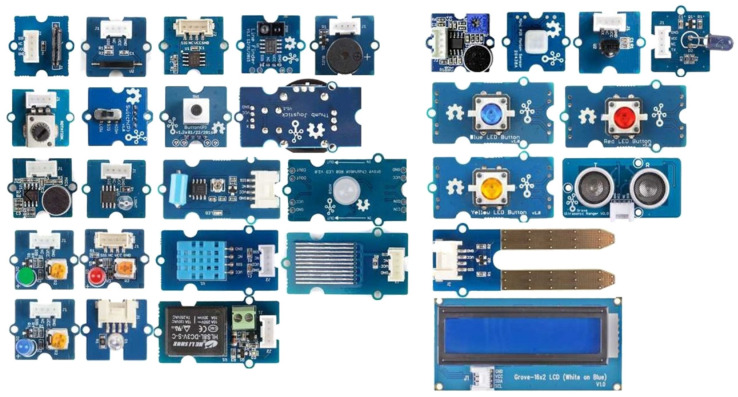
An example of Grove modules (Grove Creator kit).

Grove sensors are modular electronic sensors designed by the Seeed Studio [55]. Grove sensors include a variety of sensors, such as temperature sensors, humidity sensors, light sensors, motion sensors, sound sensors, gas sensors, and many more (Figure 3). Grove sensors use a 4-pin connector (Table 1), allowing easy plug-and-play connectivity of digital, analog, I2C, and UART Grove sensor modules (Figure 3).

STEMMA QT is a connector system developed by Adafruit Industries [56], and it represents a standardized interface for Adafruit’s wide range of STEMMA QT sensors, boards, and accessories. The STEMMA QT is designed for I2C devices and has a 4-pin connector (Table 2): (1) SCL, (2) SDA, (3) V+, and (4) GND. V+ must be between 3 V and 5 V DC; SDA and SCL are the I2C data lines. The I2C devices are expected to have pull-ups from SDA and SCL to V+.

Like STEMMA QT, Qwiic from SparkFun [57] uses 4-pin connectors for their I2C sensors, boards, and accessories. The Qwiic connector is identical to the STEMMA QT connector and uses the exact pin ordering.

**Figure 4:**
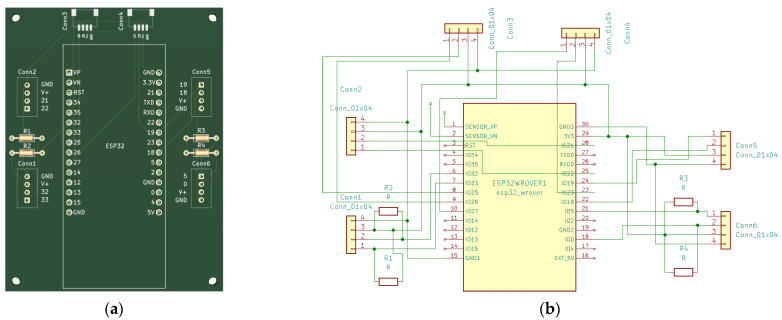
(**a**) ESP32 sensor extension board—PCB; (**b**) ESP32 sensor/actuator extension board—schematic.

**Figure 5:**
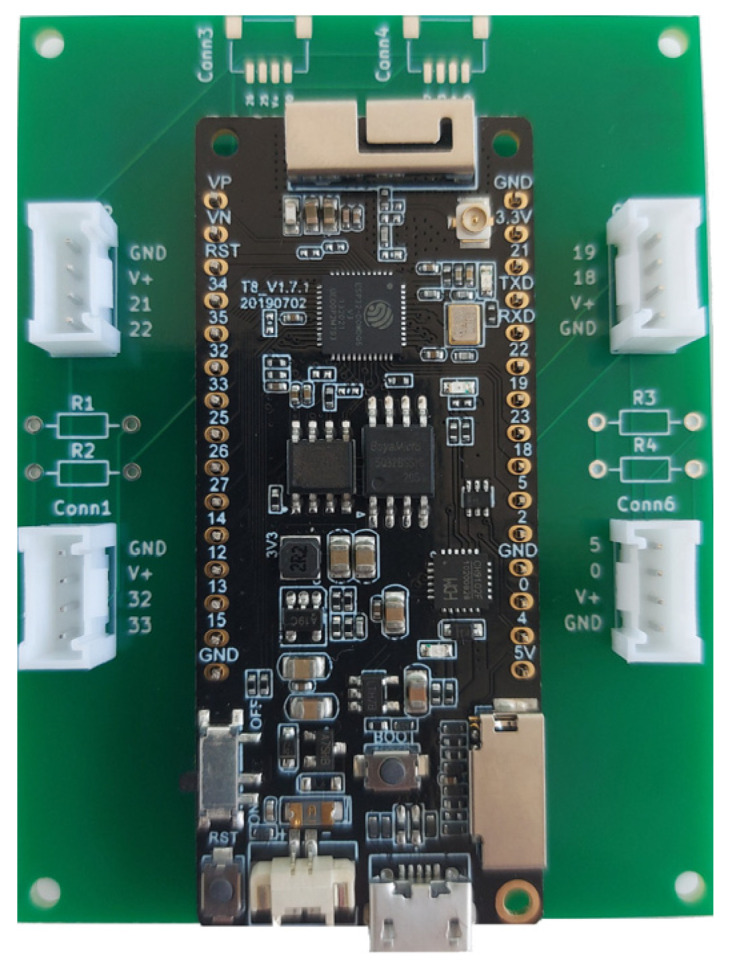
ESP32 sensor extension board.

**Figure 6:**
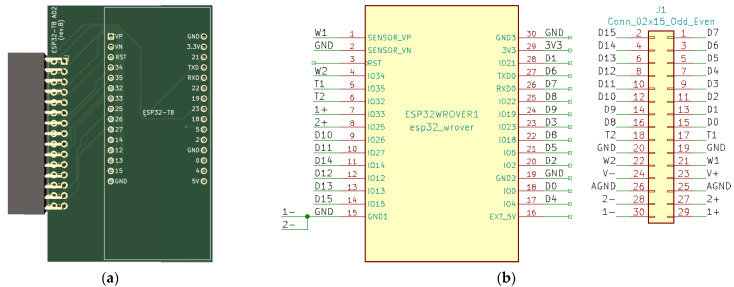
(**a**) ESP32 Analog Discovery connection board—PCB; (**b**) ESP32 Analog Discovery connection board—schematic.

**Figure 7:**
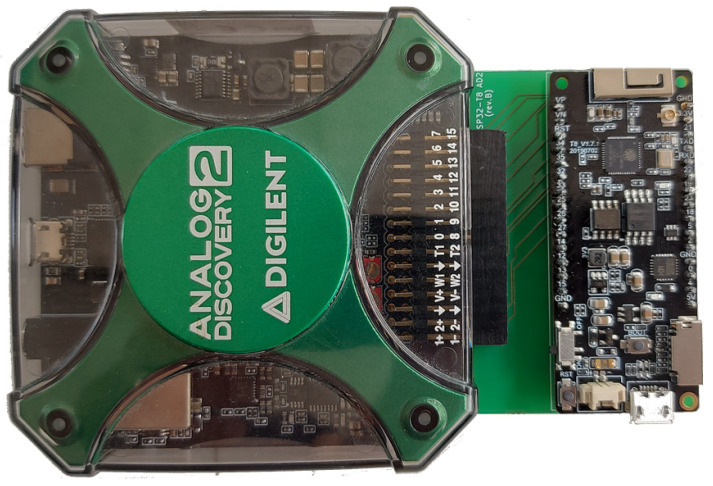
ESP32 Analog Discovery interface board connected to Analog Discovery.

For easy connection of Grove, STEMMA QT, and Qwiic sensors to the LilyGO TTGO T8 module, an expansion board (Figure 4) that contains 4 Grove (Conn1, Conn2, Conn5, and Conn6) and 2 STEMMA QT/Qwiic connectors (Conn4 and Conn5) has been developed (Figure 4 and Figure 5). For digital and analog I/O testing, an additional expansion board has been designed (Figure 6 and Figure 7) that connects LilyGO TTGO T8 to the Analog Discovery instrument.

### 3.2. Software

#### 3.2.1. Arduino IDE and the ESP32 Libraries

The Arduino IDE (integrated development environment) software application provides a platform for writing, compiling, and uploading code to Arduino-supported boards. The Arduino IDE provides a user-friendly interface and includes a (1) code editor, (2) library manager, (3) board manager, (4) serial monitor, and (5) compile and upload tools. The editor is used for writing Arduino sketches or programs. It provides syntax highlighting, auto-indentation, and other features to help write and organize code. The library manager allows the user to manage additional libraries in Arduino projects. Libraries provide additional functionality that enables easy integration of various sensors, actuators, and other components. The board manager allows the installation and management of additional Arduino board definitions. Arduino IDE supports various boards, including official boards like Arduino Uno and Arduino Mega, and third-party boards like ESP32. The serial monitor is a built-in tool that enables communication with a connected Arduino board using the serial port. The serial monitor facilitates the transmitting of data between the PC and connected Arduino hardware. A serial monitor is used mainly for debugging purposes. The compile and upload functionality compiles the code into machine language and uploads it to the board using a serial/USB connection.

The Arduino IDE has a large and active community of users with extensive online documentation, tutorials, and forums. The active open-source community contributes to Arduino development, and it provides extensive resources, libraries (Table 3), and example projects. This community support makes it easier for developers to find help, collaborate, and build upon existing projects.

#### 3.2.2. WaveForms

**Figure 8:**
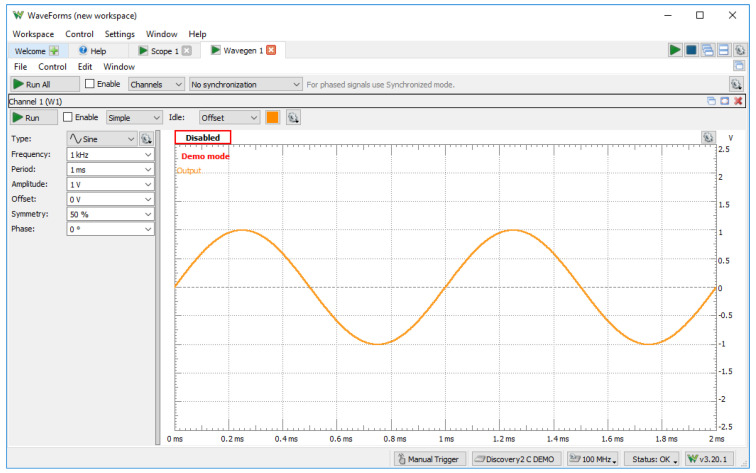
WaveForms.

WaveForms (Figure 8) is a software suite developed by the Digilent company. WaveForms is a powerful software package that complements the Analog Discovery hardware (Analog Discovery, Analog Discovery 2, Analog Discovery 3, Analog Discovery Pro 3000 Series, Analog Discovery Pro ADP5250) by providing a comprehensive set of tools for capturing, analyzing, and generating analog and digital signals. WaveForms provides a user-friendly interface with intuitive controls that allows the user to control and interact with the Analog Discovery hardware and to perform various measurements and analyses on analog and digital signals.

### 3.3. ESP32 Introductory Course

As part of the project, students first undertake an introductory course to become familiar with the ESP32 controller, architecture, periphery, and programming. Programming is written using the ARDUINO development environment, with the add-on board support for ESP32 microcontrollers. Students are introduced to the ARDUINO IDE development environment, the Analog Discovery 2 instrument capabilities, and the WaveForms software environment.

**Figure 9:**
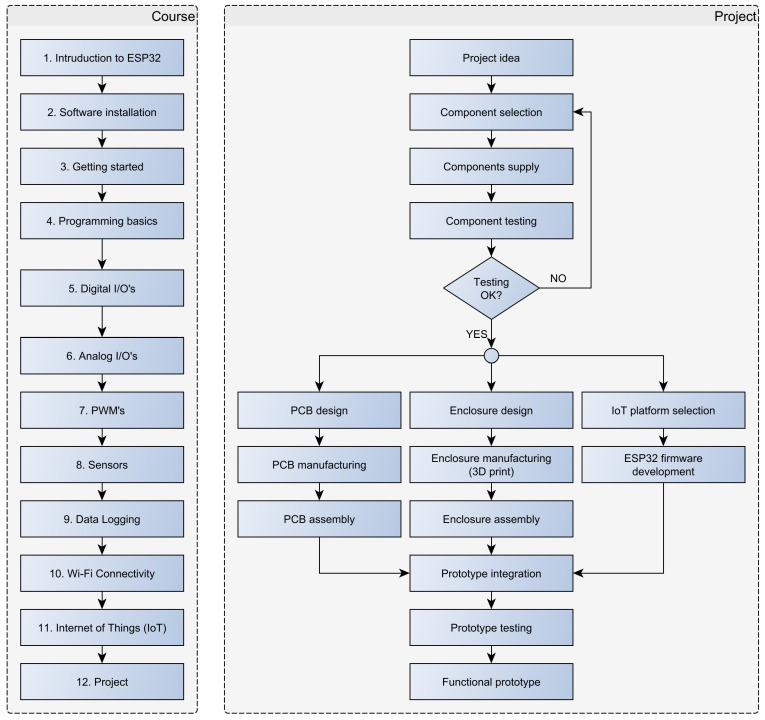
ESP32 introductory course and project flowchart.

The introductory course includes the following aspects (Figure 9):

- Introduction to ESP32: students are introduced to the ESP32 microcontroller, its features, components, and applications.
- Software installation: students install the Arduino IDE, the ESP32 board support package for Arduino IDE, and USB drivers on their PCs.
- Getting started: students connect the ESP32 module to PC, deploy (upload) simple demo programs, and monitor variables in the serial monitor.
- Programming basics: students are introduced to the C programming language in a separate subject; therefore, only a summary of the C programming language (variables, data types, operators, program structures, and functions) is given in this part.
- GPIO (general purpose inputs and outputs): students perform simple algorithms to test digital inputs and outputs. In this part, they learn how to (1) configure GPIO pins as inputs or outputs, (2) use pull-up and pull-down resistors to stabilize input readings, and (3) read and write digital pins.
- Analog I/O: in this part, students perform programs to acquire analog inputs and set analog outputs. The ESP32 module is connected to Analog Discovery 2 using the developed and presented interface board. Using an Analog Discovery Waveform generator, students create stimulus signals and perform ESP32 programs to acquire the signals using analog inputs.
- PWM (pulse width modulation): students implement algorithms that generate a PWM signal on selected output pins, and they perform the acquisition of a generated signal using Analog Discovery 2 scope.
- Sensors and actuators: in this part, students connect the ESP32 module to the developed sensor expansion board, and then they create programs to retrieve data from connected Grove, STEMMA QT, or Qwiic sensors. In addition, students learn how to install and use open-source libraries.
- Data logging: students implement algorithms that store the acquired sensor data on the SD card.
- Wi-Fi connectivity: students learn how to create an access point using the ESP32 controller, and how to connect to an existing Wi-Fi network.
- Internet of Things (IoT): students work with selected IoT platforms and build IoT applications that interact with cloud services.
- Project: students combine their skills and knowledge gained throughout the course to build various projects, such as a weather station, home automation system, or a simple robot.

After the introductory part, the students have to design their ESP32 IoT device. The students must design the device, program it, and build a suitable enclosure. Some examples of designed and developed IoT devices are described in the following section.

## 4. Results

In the first part of the project, the students become familiar with the ESP32 hardware and programming of the ESP32 controller, while in the second part, they must design their ESP32 IoT device. In most cases, the students are divided into groups of three. Each student primarily takes on one of the following tasks: (1) the design of a printed circuit board (PCB); (2) the creation of an ESP32 microcontroller application; (3) the creation of a mechanical part, which includes mainly the design and 3D printing of a device chassis.

### 4.1. Beehive Monitoring System

In addition to caring for bees, bottling the honey is part of a beekeeper’s job. A beekeeper chooses to extract honey when there is enough nectar for the colony in the nearby forests and meadows. The mass of the hive is measured in order to determine the honey content of the surrounding area. If the mass has increased since the last measurement, the beekeeper concludes that the surrounding area is honey rich and then determines the appropriate time for honey extraction. If the hive mass decreases, the beekeeper feeds the colony with sugar cake or sweetened water. A rapid decrease in weight in a short period indicates that the colony is swarming. Swarming is a phenomenon that occurs when, due to a lack of space or food, the colony moves out of the hive. For this reason, it is beneficial for the beekeeper to always have hive weight data available. Under normal conditions, a weight check of the hive should be carried out at least weekly, but this usually takes considerable time, due to the distance between the beehive and the dwelling. A system that measures the weight of the hive and other physical quantities and then presents this data to the beekeeper in a transparent and accessible way could make the beekeeper’s job much more manageable.

**Figure 10:**
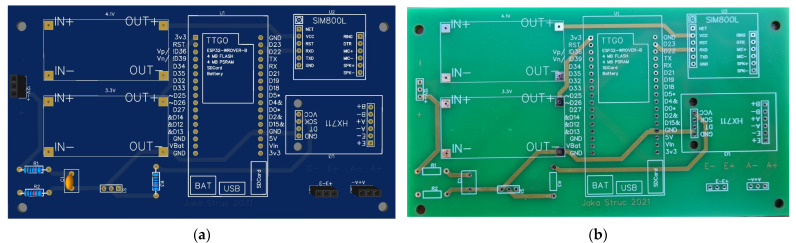
(**a**) PCB design; (**b**) manufactured PCB.

**Figure 11:**
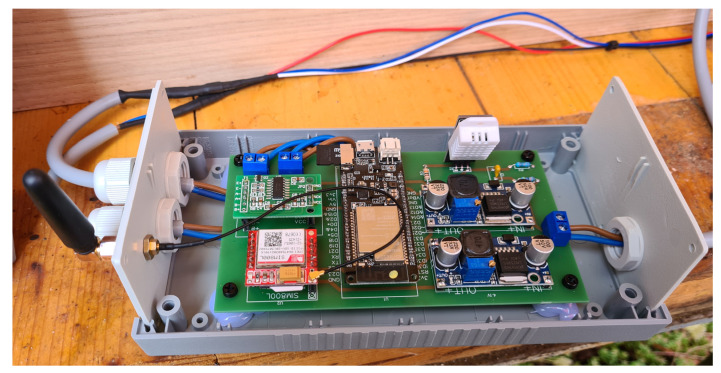
Beehive monitoring system.

**Figure 12:**
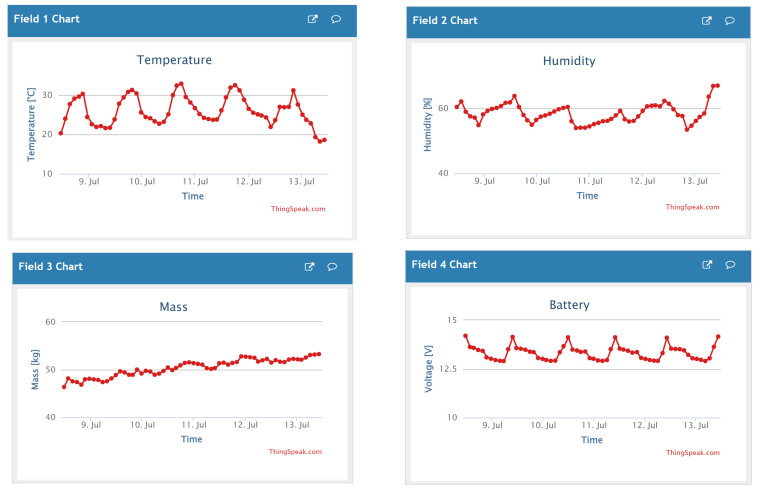
Data view in the ThingSpeak platform.

In this project, the students designed and implemented a measurement system that acquires hive weight, temperature, and ambient humidity and then saves the captured data locally on a secure digital (SD) memory card (Figure 10 and Figure 11). Using the Internet of Things (IoT), data are sent at the desired time interval to an online platform, where they are available to the user—the beekeeper—in graphical form (Figure 12). This system was installed in an existing beehive with no electricity access. A solar cell generated electricity, feeding a lead–acid battery via a power controller. Various sensors were used to measure mass, humidity, and temperature. At a configurable time interval, the measurement system connects to the mobile network and transmits the data to the selected web platform’s database.

**Figure 13:**
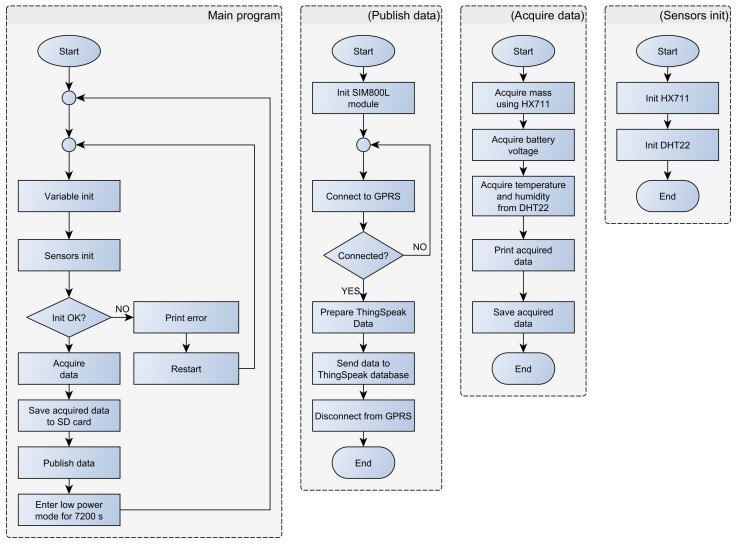
Beehive monitoring system flowchart.

The developed system is based on the TTGO T8 module. Mass measurement is implemented using the HX711 sensor and four 50 kg load cells, while the temperature and humidity measurement is achieved using the DHT22 sensor and DHT support libraries. The SIM800L module, which connects to the mobile GPRS network, is used for sending data to the ThingSpeak online database. The SIM800L module is controlled by ESP32 using a serial UART connection and AT commands. The developed measurement system is mounted in a robust ABS plastic enclosure with the dimensions 190 × 100 × 60 mm. In order to reduce power consumption, a low-power mode has been implemented on the ESP32 microcontroller. Special ESP32 functions have been utilized for this purpose. The algorithm is implemented in a setup loop, i.e., without an endless loop. The implemented algorithm performs the following (Figure 13): (1) initialization of local/global variables; (2) initialization of sensors; (3) acquisition of temperature, humidity, mass, and battery voltage; (4) logging of the acquired data to the SD card; (5) sending the acquired data to the ThingSpeak online database; (6) switching the microcontroller into low-power mode and triggering the restart timer. In low-power mode, the average current consumption is approximately 22 mA. The highest current consumption is achieved during data transfer, since the data are transmitted using a SIM module, which is energy intensive. During data transmission, the average current consumption is around 51 mA. The developed system sends data to the ThingSpeak database every 2 h (7200 s). Establishing communication and transferring data takes about 90 s, representing roughly 1% of the microcontroller’s time in low-power mode. Therefore, the average system power consumption is approximately 0.27 W (at 12 V power supply).

### 4.2. Wine Fermentation Monitoring Device

This device is designed for winemakers, and it allows the fermentation of must to be monitored remotely. During the fermentation process, winemakers have to pay attention to several quantities that subsequently influence the quality of the wine. The most important quantities to monitor are the temperature of the must and its pH; however, monitoring temperature and air quality in the wine cellar is also essential. The must’s temperature is monitored, especially at the beginning of fermentation, as it is necessary to reach the optimum temperature for the activity of the yeasts, which is between 30 °C and 36 °C.

**Figure 14:**
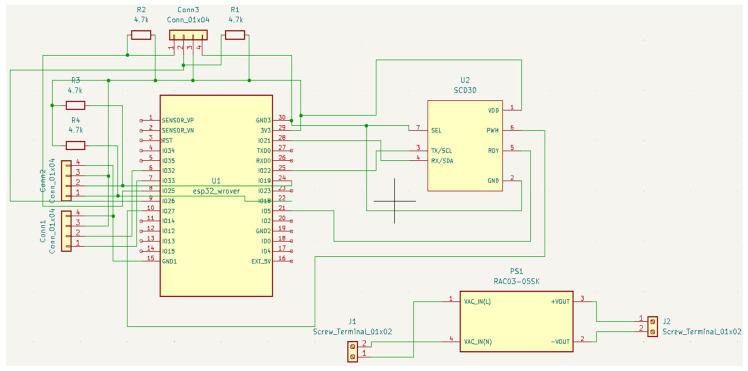
Wine fermentation monitoring device—schematic.

**Figure 15:**
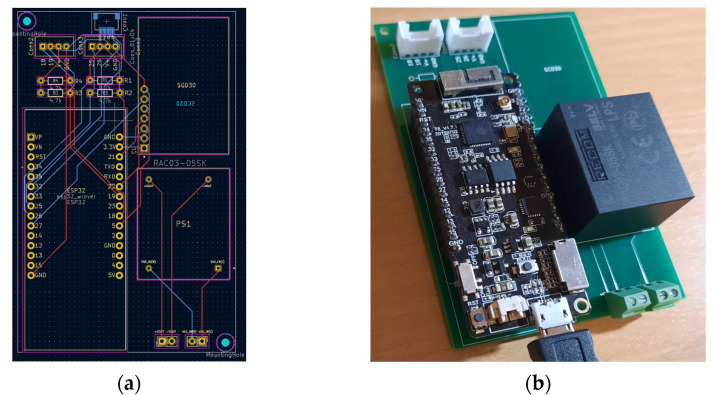
Wine fermentation monitoring device. (**a**) PCB; (**b**) manufactured device.

**Figure 16:**
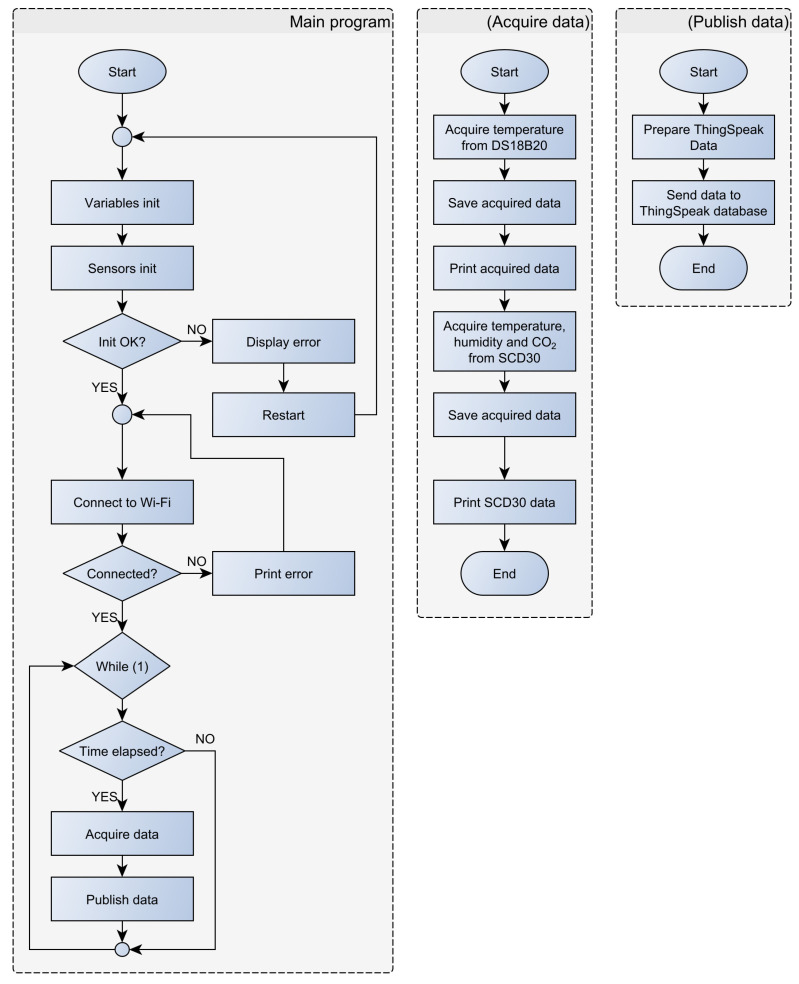
Wine fermentation monitoring system flowchart.

**Figure 17:**
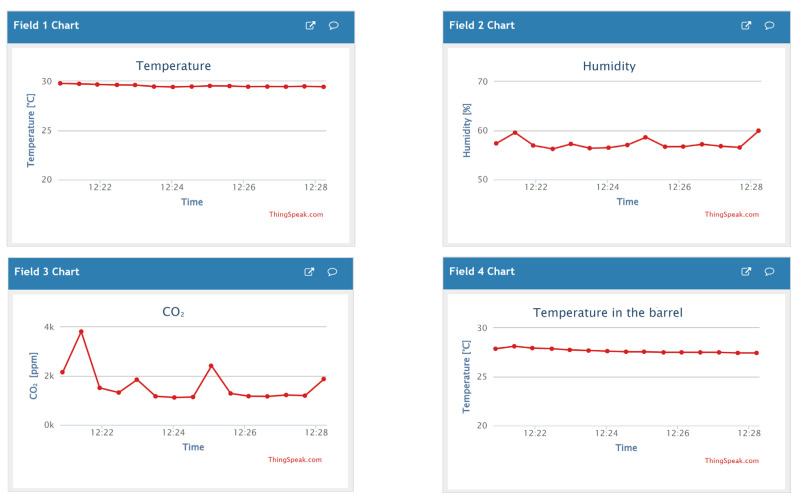
Wine fermentation monitoring results in the ThingSpeak platform.

The developed device (Figure 14 and Figure 15) acquires the temperature of the wine in the barrel and the temperature, humidity, and CO_2_ concentration in its surroundings, i.e., the wine cellar. Two sensors are used in the system. One measures the temperature of the wine in the barrel (DS18B20), and the other measures the temperature, humidity, and CO_2_ concentration in the device surroundings (SCD30). The implemented algorithm (Figure 16), after the (1) initialization of local/global variables, (2) initialization of sensors, and (3) connection to existing Wi-Fi Network, enters into the endless loop and then periodically performs the following: (1) acquires the temperature in the barrel and the temperature, humidity, and CO_2_ in the wine cellar and (2) sends the acquired data to the ThingSpeak online platform (Figure 17). Within the project, the students designed and produced a printed circuit board that contains all the necessary electronic components of the measuring device. They have also designed and manufactured a 3D-printed chassis that meets the requirements of the measurement system.

## 5. Conclusions

This paper presents a prototyping system that was designed for educational purposes. The presented solution facilitates easy connection and testing of commercially available sensors with a Grove, STEMMA QT, or Qwiic interface. The solution is compact and can be used by students at school and at home. The connection of the ESP32 module to the Analog Discovery instrument enables advanced testing of the microcontroller’s I/O periphery. The presented solution is used with mechatronics students to learn about microcontrollers, printed circuit board design, 3D design, and IoT technology. With the presented equipment, the students are introduced to some essential IoT operations, and then they have to design, develop, and implement a measurement system that acquires and sends data to the web-based platform. The solution promotes active learning and supports educators in the IoT teaching process. This article presents only two of the many projects developed by the students. However, it should be stressed that the primary purpose is to introduce students to the above-stated technologies, not to optimize the created products. In the presented course, students become familiar with IoT and basic printed circuit board design concepts. During further study, some students extend the knowledge gained with a Diploma Thesis, which usually involves the design of a more complex printed circuit board and the implementation of advanced algorithms.

## Figures and Tables

**Table 1:** Grove connector pinout.

Pin	Digital	Analog	I2C	UART
1	D0 (Primary input/output)	A0 (Primary input)	SCL (I2C Clock)	RX (UART Receive)
2	D1 (Secondary input/output)	A1 (Secondary input)	SDA (I2C Data)	TX (UART Transmit)
3	VCC (Power for Grove Module, 5 V/3.3 V)			
4	GND (Ground)			

**Table 2:** STEMMA QT and Qwiic pinout.

Pin	I2C
1	SCL (I2C Clock)
2	SDA (I2C Data)
3	V+ (Power for STEMMA QT/Qwiic modules 5 V/3.3 V)
4	GND (Ground)

**Table 3:** List of some Arduino ESP2 open-source community libraries.

Library Group	Libraries
Wi-Fi	WiFiManager, ESPAsyncWiFiManager, WiFiEsp, WiFiNINA
MQTT	PubSubClient, Adafruit MQTT Library, MQTT-TLS, MQTT-SN
Display	Adafruit SSD1306, Adafruit GFX, TFT_eSPI, U8g2, ILI9341
Sensors	Adafruit BME280, DHT sensor library, Adafruit MCP23017, BH1750
Web Server	ESPAsyncWebServer, AsyncTCP, ESP8266WebServer, ESP8266HTTPClient
JSON	ArduinoJson, ArduinoJson5, ESPJson, JsonStreamingParser
LED Control	Adafruit NeoPixel, FastLED, WS2812FX
Bluetooth	BLEPeripheral, BLEScan, ESP32 BLE Arduino, ESP32 Bluetooth
SD Card	SD, SPIFFS, SdFat, ESP32_SD_Card, SD_MMC
MQTT-based IoT	Blynk, Adafruit IO, Cayenne, Ubidots, Losant
Motor Control	AccelStepper, ESP32Servo, DRV8825, Adafruit Motor Shield
Real-Time Clock	DS3231, NTPClient, Time, Timezone

## Data Availability

Not applicable.
